# Effects of Methotrexate Therapy on the Levels of Gonadotropic Hormones in Rheumatoid Arthritis Patients of Reproductive Age

**DOI:** 10.7759/cureus.7632

**Published:** 2020-04-11

**Authors:** Min Tun Kyaw, Rajalingham Sakthiswary, Zainudin Ani Amelia, Abdul Rahman Rahana, Md Mansor Munirah

**Affiliations:** 1 Obstetrics and Gynaecology, Universiti Kebangsaan Malaysia Medical Centre, Kuala Lumpur, MYS; 2 Internal Medicine: Rheumatology, Universiti Kebangsaan Malaysia Medical Centre, Kuala Lumpur, MYS; 3 Chemical Pathology, Universiti Kebangsaan Malaysia Medical Centre, Kuala Lumpur, MYS

**Keywords:** menopause, rheumatoid arthritis, methotrexate, follicular stimulating hormone

## Abstract

Background: Methotrexate (MTX), which is the anchor drug in rheumatoid arthritis (RA), targets actively proliferating cells including the oocytes and granulosa cells which may impair the ovarian reserve. The purpose of this study was to determine the effects of MTX therapy on gonadotropic hormones, i.e. follicular stimulating hormone (FSH) and luteinizing hormone (LH) in female RA patients of reproductive age.

Materials and methods: This is a cross-sectional study conducted at the Universiti Kebangsaan Malaysia Medical Centre (UKMMC), from January 2018 to July 2018. Women with RA aged between 15 and 49 years who were on MTX therapy for at least six months, were consecutively recruited. All subjects were interviewed to gather information on their menstrual history and menopausal symptoms. The medical records were reviewed to obtain further data on the disease characteristics and RA treatment. The RA disease activity was determined using the DAS 28 scoring system. All subjects were tested for their serum FSH and LH levels.

Results: A total of 40 patients were included in this study. The median dose of MTX used by the subjects was 12.5 mg weekly. The mean cumulative MTX dose was 1664.92 ± 738.61 mg. More than half (53.1%) of the subjects reported menopausal symptoms especially hot flushes. We found that FSH levels had a significant positive correlation with cumulative MTX dose [(r = 0.86), p < 0.001] and the duration of MTX therapy [(r = 0.84), p < 0.001]. Besides, there was a significant relationship between disease activity based on DAS 28 and FSH levels (p < 0.01). Age, body mass index, disease duration, and weekly MTX dose showed no associations with the FSH levels. On multivariate analysis, DAS 28 was found to be the only parameter that remained significant [β = 1.74 (95% CI 1.17-2.31), p < 0.001]. The LH levels, on the other hand, were not associated with MTX therapy or disease activity.

Conclusion: Higher levels of FSH, which is an indicator of diminished ovarian reserve, have a significant positive relationship with disease activity, cumulative dose, and duration of MTX therapy in RA.

## Introduction

Rheumatoid arthritis (RA) predominantly affects women with a sizable proportion of them being of reproductive age, i.e. between 15 and 49 years [1]. RA and its treatment may interfere with the female reproductive physiology. The vast majority of patients with RA are treated with methotrexate (MTX) which is a folate antagonist that inhibits DNA synthesis. MTX targets actively proliferating cells including the oocytes and granulosa cells [2] which may impair the ovarian reserve. High doses of MTX used in oncology patients are known to cause premature ovarian failure (POF) or early menopause. Postchemotherapeutic menopause has been reported in up to 68% of breast cancer survivors treated with drug regimens that included high doses of MTX [3].

The MTX dose used in RA is much lower than in cancer treatment. Dosages range from 5 to 25 mg/week in RA whereas may reach up to 1,000 mg/m2 of body surface area in certain types of malignancies. The effects of MTX therapy on ovarian function at lower doses are not well-proven and remain to be elucidated. An animal study conducted by Karri et al. showed that MTX given to rats over a period of 20 days leads to a reduction of serum progesterone and estradiol levels. There were several morphological and histological changes observed in the reproductive organs of the MTX-treated rats in a dose-dependent fashion. There was a reduction in the weight of the ovaries, reduction in the pre-antral and antral follicular growth, and suppression of ovarian steroidogenesis. These alterations caused raised gonadotropin levels, i.e. serum follicular stimulating hormone (FSH) and luteinizing hormone (LH) [4] .

Premature ovarian failure or early menopause may result from MTX therapy due to accelerated depletion of the ovarian reserve secondary to direct primordial follicle damage. POF is defined as the cessation of menses associated with secondary amenorrhea, sex steroid deficiency, and elevated serum levels of gonadotropins before the age of 40 [5]. Beyond infertility, POF has other health implications such as coronary artery disease, depression, and osteoporosis [6].

Few reports indicated an earlier age of natural menopause in women with RA as compared to the general population. Besides, lower parity and longer mean time to achieve a spontaneous pregnancy suggest diminished ovarian reserve in RA [7]. The occurrence of menopause at a younger age in RA patients is probably multifactorial. It may be the consequence of gonadotoxicity induced by MTX therapy and from an antibody-mediated ovarian injury.

The purpose of this study was to investigate the effects of MTX therapy on the gonadotropin levels in RA. Basal FSH is the most widely used blood marker in the evaluation of ovarian reserve [8].

## Materials and methods

Study population and design

This is a cross-sectional and observational study conducted in a tertiary teaching hospital in Malaysia (Universiti Kebangsaan Malaysia Medical Centre, UKMMC). We recruited women with RA of reproductive age (between 15 and 49 years according to the World Health Organization), who were on MTX therapy for a minimum period of six months. Patients who were on hormone replacement therapy, hormonal contraception, history of gynecological malignancy or surgical menopause were excluded. This study was approved by the Medical Research and Ethics Committee of UKMMC (Project Code: FF-2018-138).

All subjects were interviewed to gather information pertaining to their menstrual history and symptoms of POF (amenorrhea, hot flushes, night sweats, vaginal dryness, irritability, and decreased sexual desire). The medical records were reviewed to determine the RA disease characteristics, MTX dose, and treatment duration. The disease activity of all subjects was assessed using the DAS28-ESR (disease activity score based on 28 joints using the erythrocyte sedimentation rate). Besides, the subjects were tested for their serum FSH and LH levels.

Follicle stimulating hormone (FSH) and luteinizing hormone (LH) measurement

The blood samples of the subjects were collected and centrifuged at 3000g for 10 min. The plasma was stored at -70°C for the measurement of FSH and LH concentrations. The gonadotropins were tested using a chemiluminescence assay kit (Abbott Architect, Abbott, IL, USA).

Statistical analysis

Data analysis was performed using SPSS (Statistical Package of Social Science) version 23 (SPSS, IBM Corporation, NY, USA). The relationship between the gonadotropins and the clinical variables such as MTX duration, cumulative MTX dose, disease duration, and disease activity was determined using the bivariate correlation analysis. Multivariate analysis was performed as an extension of the bivariate analyses using the significant variables from the latter to predict the gonadotropin levels. The p values less than 0.05 were considered statistically significant.

## Results

Clinical characteristics of study population

A total of 66 RA patients were eligible to participate in this study, of whom 40 agreed to participate. Due to missing data, only 32 subjects were included in the data analysis. Table 1 summarizes the demographic and clinical characteristics of the study population. The mean age of the study population was 35.22 ± 5.85 years. Majority (75%) of them were married parous women. The mean disease duration was approximately four years (48.72 ± 34.46 months). Although MTX is the first line disease modifying anti-rheumatic drug (DMARD) in RA, the mean duration on MTX therapy was shorter than the disease duration as MTX therapy was either deferred or interrupted in 18 (56.3%) subjects who wanted to conceive at some stage of their illness. The highest dose of MTX used in this study was 15 mg weekly. Many of the subjects could not tolerate higher doses due to gastrointestinal side effects.

**Table 1:** Clinical characteristics of study population. Values are expressed as number (%)*, median (range)**, and mean±standard deviation. MTX, methotrexate; ESR, erythrocyte sedimentation rate; CRP, C-reactive protein

Variables	n=32
Age (years)	35.22 ± 5.85
Ethnicity* Chinese Malays Indians	5 (15.6) 26 (81.1) 1 (3.1)
Seropositive*	24 (75.0)
Body mass index (kg/m^2^)	27.47 ± 5.32
Disease duration (months)	48.72 ± 34.46
Duration on MTX (months)	36.41 ± 26.22
Disease activity score (DAS 28)	3.05 ± 1.21
ESR (mm/h)	44.69 ± 22.41
CRP (mg/dL)	0.99 ± 1.75
MTX dose (mg/week)**	12.5 (5.0 - 15.0)
Cumulative MTX dose (mg)	1664.92 ± 738.61

The mean disease activity based on DAS 28 was low disease activity (DAS 28 between 2.6 and 3.2). Many of the patients (43.8%) were on combination DMARD therapy especially with conventional DMARDS such as sulfasalazine, hydroxychloroquine, and leflunomide. The steroid dose of all the subjects was below 5 mg daily.

All patients in our study had attained menarche with majority (84.4%) of them having regular menses of 21-35 days cycle. One of the subjects had underlying polycystic ovarian syndrome with oligomenorrhoea. More than half of the study population had menopausal symptoms with hot flushes being the most common symptom (46.9%). The subjects with menopausal symptoms had a significantly higher cumulative dose of MTX as compared to the asymptomatic ones (p < 0.05). The mean values of FSH (4.78 ± 2.64 mIU/mL) and LH (5.77 ± 6.59 mIU/mL) were within normal limits; not suggestive of ovarian insufficiency. None of the subjects had FSH levels in the menopausal range (above 30 mIU/mL).

Correlation between the gonadotrophic hormones and the clinical covariates

We found that the FSH levels had a significant correlation with DAS 28, cumulative MTX dose, and duration of MTX treatment (p values of <0.05 for all of the above correlations) (Table 2). The relationship between FSH levels and the aforementioned variables was strong as the correlation coefficient was above 0.4. However, on multivariate analysis, DAS 28 was the only predictor variable which remained significant [β = 1.74 (95% CI 1.17- 2.31), p = < 0.05] when the effect of the other predictors was held constant. As opposed to the FSH levels, the LH levels had no significant correlation with the clinical parameters.

 

**Table 2:** Correlation between serum FSH levels and clinical variables. DAS 28, 28 joint based disease activity score; CRP, C-reactive protein; ESR, erythrocyte sedimentation rate; FSH, follicular stimulating hormone The test was performed using Spearman’s rank correlation test.

Values	Serum FSH		
	r_s_	r^2^	p
Age	0.89	0.01	0.63
ESR	0.02	0.00	0.92
CRP	0.08	0.01	0.68
DAS 28	0.94	0.89	<0.05
Disease duration	0.04	0.00	0.82
MTX treatment duration	0.84	0.71	<0.05
Cumulative MTX dose	0.86	0.74	<0.05

## Discussion

The novel findings of this study include the significant correlation of the FSH levels with the cumulative MTX dose, duration of MTX therapy, and the DAS 28 scores. This study highlights that long-term low dose MTX therapy in RA may have effects on ovarian function. Although frank ovarian failure did not occur, subjects with exposure to higher doses of MTX were more prone to have menopausal symptoms. Menopausal symptoms are known to occur for years before complete cessation of menses. Sherman et al. noted a monotropic rise in FSH levels despite normal menstrual cycles during the menopause transition [9]. The FSH levels tend to increase progressively with age due to the loss of ovarian inhibin B [10]. In the early menopausal transition, there are raised levels of FSH with normal levels of estrogen [11]. This may explain the regular menstruations in the majority of our subjects.

 Our findings were in contrast with many of the published studies in the literature. In women who were treated with MTX for ectopic pregnancy, there were no differences in the FSH levels, antral follicle count, oocytes retrieved, or fertilization rate between pre- and post-MTX therapy. The cumulative dose of MTX in ectopic pregnancies was much lower than in this study [12-13]. Banas et al. [14] reported that RA patients underwent menopause at a significantly younger age than healthy women but found no association with MTX treatment [14]. A strict comparison cannot be made with the above-mentioned study as cumulative MTX dose and gonadotropin levels were not included. Brouwer et al. [15] reported that serum anti-Mullerian hormone (AMH), a marker for ovarian reserve was not affected by either disease activity or short-term use of MTX for six months. The subjects in the study by Brouwer et al. were exposed to a much lower cumulative dose of MTX compared to our subjects [15]. Bower et al., in contrast to the above-mentioned studies, disclosed that MTX therapy in gestational trophoblastic tumors hastened menopause by three years on an average [16]. In a study involving systemic lupus erythematosus subjects [17], there was a significant inverse correlation between AMH levels and cumulative dose of MTX, parallel to our findings.

The aforementioned study was the first to identify cumulative MTX dose as a predictor of ovarian insufficiency in an autoimmune disease. Taken together, long-term use of MTX although at low doses, may increase the likelihood of developing early menopause or POF. The effects of MTX on ovarian reserve are probably dose-dependent.

 There is a profound lack of human studies looking into the relationship between cumulative MTX dose and ovarian reserve. Murine studies have identified damage to the primordial follicles following MTX therapy [4, 18]. The underlying mechanism of MTX-related ovarian damage is poorly understood. The enzyme dihydrofolate reductase (DHFR) which reduces folic acid to tetrahydrofolic acid is inhibited by MTX. This interferes with the synthesis of purines and the conversion of deoxyuridylate to thymidylate in the synthesis of DNA for cellular growth. Animal studies have shown that MTX therapy causes cells to abruptly arrest in metaphase [19].

 Disease activity was the only clinical variable, which remained significantly associated with the FSH levels on multivariate analysis. Systemic inflammation in RA may affect a woman’s overall health status. This is in keeping with studies of other autoimmune conditions that have reported reduced AMH levels in women with Crohn’s disease [20] or systemic lupus erythematosus [21] compared to healthy controls. Kass et al. [22] reported that changes in FSH levels were found to correlate positively with the cytokine changes in RA. The cytokines which were studied included the key pro-inflammatory cytokines such as interleukin(IL) 1, tumor necrosis factor α, IL-2, IL-2R, IL-8, monocyte chemoattractant protein (MCP)-1, macrophage inflammatory protein (MIP)-1α, MIP-1β, and eotaxin. There is growing evidence on hormone-cytokine crosstalk in RA. This partially explains the tendency for disease activity flares during periods of increased FSH, such as the postpartum period [23].

 This study was not without limitations. The small sample size decreases the statistical power while increasing the margin of error. Therefore, the findings should be interpreted with caution. Many of the RA patients at our center were postmenopausal and hence, did not fulfil the study eligibility criteria. We used FSH as the marker of ovarian reserve mainly due to budget constraints. The FSH levels tend to vary from one day to another whereas AMH levels are more stable and consistent. AMH is a more reliable biomarker of ovarian reserve as it correlates better with the antral follicle count compared to FSH [24].

## Conclusions

Our findings suggest that the FSH level in RA is influenced by the disease activity and cumulative MTX dose. Higher cumulative doses of MTX and disease activity may increase the risk of early menopause or POF. Female RA patients should be comprehensively assessed for their ovarian function as POF has deleterious effects on cognition, mood, cardiovascular, and bone health.
